# Comparative Diagnostic Accuracy of Clinical Pallor at Different Anatomical Sites for Detecting Anemia in Pregnant Women Using HemoCue® as the Reference Standard

**DOI:** 10.7759/cureus.114293

**Published:** 2026-08-10

**Authors:** Dhivagar Jegadeesan, Vijaykumar Rajendran, Saranya Rajamanickam

**Affiliations:** 1 Community Medicine, Swamy Vivekanandha Medical College Hospital and Research Institute, Namakkal, IND

**Keywords:** anemia, clinical pallor, diagnostic accuracy, pregnancy, primary care

## Abstract

Background

Anemia during pregnancy remains a major public health problem in low-resource settings. Although hemoglobin estimation using portable devices such as the HemoCue® Hb 201 System (HemoCue AB, Ängelholm, Sweden) is reliable, clinical assessment of pallor continues to be widely used in primary care because of its simplicity, feasibility, and low cost. However, evidence comparing the diagnostic accuracy of pallor at different anatomical sites among pregnant women is limited.

Objective

This study aimed to determine and compare the diagnostic accuracy of clinical pallor at different anatomical sites for detecting anemia among pregnant women using HemoCue® hemoglobin estimation as the reference standard.

Methods

A cross-sectional diagnostic accuracy study was conducted among 186 pregnant women attending the Rural Health Training Centre (RHTC) in Barwala, Delhi. Clinical pallor was assessed under natural light at the palpebral conjunctiva, nail bed, palm, and palmar crease. Hemoglobin concentration was measured using HemoCue® as the reference standard. Diagnostic accuracy measures, including sensitivity, specificity, positive predictive value (PPV), negative predictive value (NPV), likelihood ratios, and Cohen's kappa (κ), were calculated for detecting anemia (hemoglobin <11 g/dL).

Results

Among the 186 pregnant women enrolled, 139 (74.7%) were anemic based on HemoCue® hemoglobin estimation. Palpebral conjunctival pallor demonstrated the highest sensitivity (98.6%) and specificity (93.6%), with excellent agreement (κ = 0.92). Nail bed pallor showed a sensitivity of 81.3% and a specificity of 42.6%, while palmar pallor demonstrated a sensitivity of 71.9% and a specificity of 78.7%. Palmar crease pallor had the lowest sensitivity (46.0%) but a specificity of 78.7%. Palpebral conjunctival pallor also showed the highest positive LR (LR+; 15.45) and the lowest negative LR(LR-; 0.02).

Conclusion

Palpebral conjunctival pallor demonstrated the highest diagnostic accuracy for detecting anemia in pregnant women and may serve as a reliable screening method in primary care settings where hemoglobin estimation is unavailable. Palm and palmar crease examination may provide complementary clinical information but are less accurate than conjunctival assessment.

## Introduction

Anemia in pregnancy is defined by the World Health Organization (WHO) as a hemoglobin concentration below 11.0 g/dL [1]. It remains a major public health problem worldwide. According to the WHO, the global prevalence of anemia among pregnant women was 35.5% in 2023, with a substantially higher burden in low- and middle-income countries than in high-income countries [2].

The Southeast Asian region bears a disproportionately high burden of maternal anemia, with a reported prevalence of 48.7% [3]. According to the National Family Health Survey (NFHS)-4, 50.3% of pregnant women in India and 46.1% in Delhi were anemic [4]. The NFHS-5 reported a prevalence of 52.2% among pregnant women in India and 42.2% in Delhi, indicating only a modest decline over time [5]. However, rural-specific estimates of anemia among pregnant women in Delhi are not available.

In India, anemia remains a major public health challenge despite the implementation of the Anaemia Mukt Bharat initiative under the National Health Mission, which aims to reduce the burden of anemia through iron supplementation, deworming, nutritional interventions, and universal screening [6]. Despite these efforts, early detection of anemia at the primary care level remains challenging because laboratory services are not consistently available in many rural and resource-constrained settings [7].

According to the WHO Antenatal Care Guidelines, assessment for pallor should be performed during routine antenatal visits to facilitate the early detection of anemia among pregnant women [8]. Pallor examination is a simple, rapid, inexpensive, and non-invasive screening method that is particularly valuable in resource-limited settings where laboratory-based hemoglobin estimation may not be feasible because of financial, logistical, or infrastructural limitations [9]. Clinical pallor can be assessed at several anatomical sites, including the palpebral conjunctiva, nail bed, palm, palmar crease, tongue, and lip mucosa, although its diagnostic accuracy varies across examination sites [10].

Several studies have evaluated the diagnostic performance of clinical pallor for detecting anemia. Sheth TN et al. demonstrated that conjunctival pallor is a useful clinical indicator of anemia, with better diagnostic performance than many other physical examination findings [11]. Bergsjø P et al. reported that clinical assessment of pallor remains valuable for screening anemia during pregnancy in resource-limited settings, although its accuracy varies according to the anatomical site examined [12]. Similarly, Kalantri A et al. found that conjunctival pallor was more accurate than pallor of the palm or nail bed and emphasized the importance of standardized examination techniques to improve diagnostic reliability [13]. Despite these findings, evidence directly comparing multiple anatomical sites for detecting anemia among pregnant women in primary healthcare settings remains limited, particularly using portable point-of-care hemoglobin estimation as the reference standard.

Identifying the anatomical site with the highest diagnostic accuracy could improve screening strategies in settings where laboratory testing is unavailable or delayed. Therefore, this study aimed to determine and compare the diagnostic accuracy of clinical pallor at four anatomical sites, the palpebral conjunctiva, nail bed, palm, and palmar crease, for detecting anemia among pregnant women using HemoCue® Hb 201 System (HemoCue® AB, Ängelholm, Sweden) hemoglobin estimation as the reference standard.

## Materials and methods

Study setting and design

This facility-based cross-sectional study was conducted at the Rural Health Training Centre (RHTC), Barwala, Delhi, India, the field practice area of the Department of Community Medicine, Maulana Azad Medical College (MAMC), New Delhi, India. Accordingly, ethical approval was obtained from the Institutional Ethics Committee of MAMC (Reference No. F.No.17/IEC/MAMC/2024/Govt. Med./02; Approval dated 27 October 2024). The study was carried out from October 2024 to October 2025. 

Sample size calculation

The sample size was calculated using Buderer's formula for estimating sensitivity in diagnostic accuracy studies. The formula is as follows: 

$n=\frac{Z^{2}_{1-\alpha/2} .Sn.(1-Sn)}{L^{2}.P}$

Based on the study by Bala DV et al. [14], sensitivity (Sn) of 91.7%, disease prevalence (P) of 69.8%, a 95% confidence level (Z = 1.96), and an absolute precision (L) of 5% were assumed.

The minimum required sample size was 167 participants. After accounting for an anticipated 10% non-response rate, the final sample size was 186 participants.

Sampling method

A non-probability consecutive sampling method was used.

Inclusion and exclusion criteria

**Figure 1:**
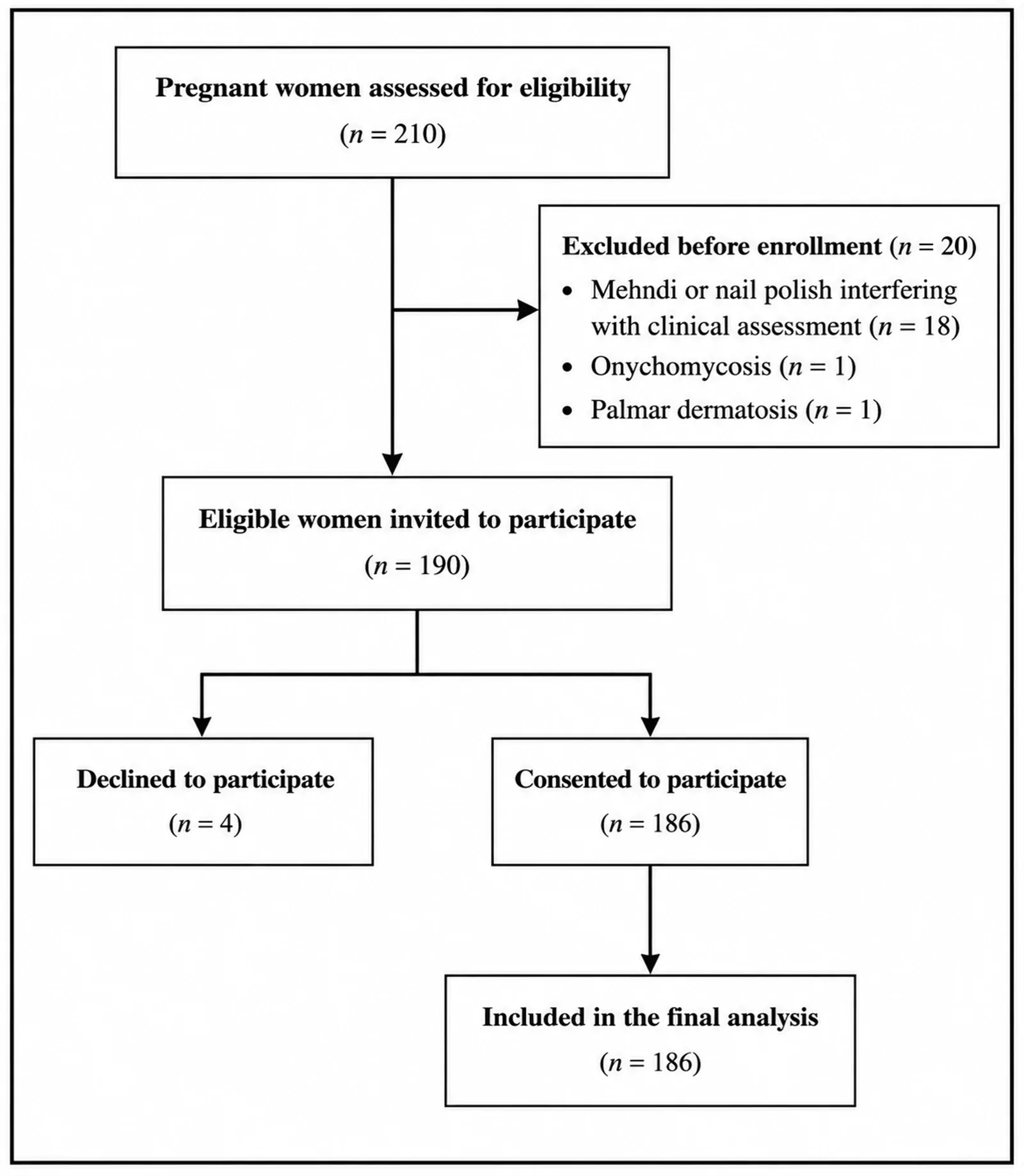
Flow Diagram of Study Participant Selection

Eligible participants included pregnant women of any trimester attending the antenatal clinic at the RHTC, Barwala, who were willing to participate, provided written informed consent, and were available for both clinical examination and hemoglobin estimation were included in the study. Pregnant women were excluded if they had mehendi (henna) on the palms or fingers, used nail polish or artificial nails that interfered with nail bed assessment, had dermatological conditions affecting the palms, nails, or conjunctiva, had acute illness or severe dehydration that could affect general appearance or peripheral perfusion, or had any condition that precluded an adequate clinical examination. The participant selection process is shown in Figure 1.

Ethical clearance

Ethical clearance was obtained from the Ethics Committee of MAMC, New Delhi, India (Reference number: F.no. 17/IEC/MAMC/2024/Govt. Med./02). Written informed consent was obtained from all participants before enrolment in the study.

Data collection method

Data were collected from pregnant women attending the antenatal clinic at the RHTC, Barwala, during the study period. Eligible participants who met the inclusion criteria were informed about the purpose and procedures of the study, and those who provided written informed consent were enrolled. A pretested semi-structured questionnaire was used as the study instrument, comprising sections on participant identification, sociodemographic characteristics, obstetric history, clinical examination, and laboratory investigations (Appendix A).

Clinical pallor is the abnormal paleness of the skin or visible mucous membranes observed on physical examination, primarily resulting from a reduced hemoglobin concentration and commonly used as a clinical sign of anemia.

Clinical examination was performed by the principal investigator under adequate natural light, with particular emphasis on detecting pallor at the lower palpebral conjunctiva, nail beds, palms, and palmar creases. The clinical assessment was conducted independently, and the investigator was blinded to the HemoCue® hemoglobin results.

To assess inter-observer reliability in the interpretation of clinical pallor, two registered medical practitioners independently examined the first 30 eligible pregnant women enrolled in the study for pallor at the palpebral conjunctiva, nail bed, palm, and palmar crease using standardized operational definitions. The interval between the two observers' examinations ranged from 15 minutes to three hours. Percentage agreement and Cohen's kappa (κ) statistic were calculated to assess inter-observer reliability. These participants were subsequently included in the final analysis. Following this calibration exercise, all clinical examinations during the remainder of the study were performed by the principal investigator to ensure consistency.

Assessment of pallor was performed using standardized operational definitions for each anatomical site. Conjunctival pallor was considered present when the anterior rim of the lower palpebral conjunctiva appeared as pale as the deeper posterior conjunctiva. Palmar pallor was defined as generalized pallor of the palm compared with the examiner's palm or that of a healthy individual under similar lighting conditions. Nail bed pallor was assessed by applying gentle pressure over the nail bed and observing the color after release; persistence of pallor with delayed return of normal color was considered indicative of pallor. Palmar crease pallor was defined as palmar creases appearing as pale as or paler than the surrounding skin and was considered suggestive of more severe anemia.

Anemia was classified according to the WHO criteria for pregnant women as mild (10.0-10.9 g/dL), moderate (7.0-9.9 g/dL), and severe (<7.0 g/dL) [1].

Hemoglobin estimation

HemoCue® hemoglobin estimation was selected as the reference standard because it is a validated point-of-care method with high accuracy and good agreement with laboratory hemoglobin estimation. It is widely used in field-based and primary care settings where laboratory facilities may not be readily available [12].

Hemoglobin concentration was measured using the HemoCue® Hb 201 System. Capillary blood was obtained by finger prick under aseptic precautions and collected into a disposable HemoCue® microcuvette according to the manufacturer's instructions. Hemoglobin concentration was measured immediately using the portable photometer. The HemoCue® Hb 201 System determines hemoglobin concentration using the azide methemoglobin method at wavelengths of 570 nm and 880 nm. The instrument was operated according to the manufacturer's recommendations and underwent daily quality checks using the manufacturer's control cuvette. All measurements were performed by trained investigators following standardized procedures. A single hemoglobin measurement was obtained for each participant.

Data Analysis

Data were entered into Microsoft Excel (Microsoft Corp., Redmond, WA, USA) and analyzed using IBM SPSS Statistics version 25.0 (IBM Corp., Armonk, NY, USA). Continuous variables were summarized as mean ± standard deviation (SD), while categorical variables were expressed as frequencies and percentages. The prevalence and severity of anemia were determined according to the WHO classification based on hemoglobin concentration. There were no missing data, and all 186 participants were included in the final analysis.

The diagnostic performance of clinical pallor at the palpebral conjunctiva, nail bed, palm, and palmar crease was evaluated using hemoglobin estimation by the HemoCue® Hb 201 System as the reference standard. Two-by-two contingency tables were constructed for each anatomical site. Diagnostic accuracy measures, including sensitivity, specificity, positive predictive value (PPV), negative predictive value (NPV), positive likelihood ratio (LR+), negative likelihood ratio (LR−), and overall diagnostic accuracy (percentage agreement), were calculated with 95% confidence intervals (95% CI). Sensitivity was defined as the proportion of participants with anemia correctly identified by clinical pallor, whereas specificity was the proportion of participants without anemia correctly identified as negative. PPV represented the probability that participants with clinical pallor truly had anemia, and NPV represented the probability that participants without clinical pallor were free of anemia. The LR+ indicated the increase in the odds of anemia following a positive clinical finding, whereas the LR− indicated the decrease in the odds of anemia following a negative clinical finding. Agreement between the clinical assessment and HemoCue® hemoglobin estimation was assessed using Cohen's κ statistic. Inter-observer reliability for the assessment of pallor at different anatomical sites was also evaluated using percentage agreement and Cohen's κ statistic.

## Results

The mean age of the participants was 24.06 ± 3.36 years. Most participants were Hindu (149, 80.1%) and belonged to the Other Backward Class (OBC) category (111, 59.7%). Nearly half of the participants had completed middle school education (92, 49.5%), and more than half were unemployed (111, 59.7%). The largest proportion of participants belonged to socioeconomic class III according to the Modified B.G. Prasad Scale (65, 34.9%) [15], lived in nuclear families (108, 58.1%), were primigravida (114, 61.3%), and were in the second trimester of pregnancy (112, 60.2%) (Table 1).

**Table 1:** Sociodemographic and Obstetric Characteristics of the Study Participants (N = 186) Data are presented as mean ± standard deviation (SD) for continuous variables and n (%) for categorical variables. SC: Scheduled Caste; ST: Scheduled Tribe; OBC: Other Backward Class. Socioeconomic status was classified according to the Modified B.G. Prasad Scale 2024 based on per capita monthly income [15]. Gravidity was categorized as primigravida (first pregnancy), gravida 2 (second pregnancy), and gravida ≥3 (third or higher pregnancy).

Characteristics	Category	Value
Age (years)		24.06 ± 3.36
Religion	Hindu	149 (80.1)
	Muslim	19 (10.2)
	Others	18 (9.7)
Caste	SC	38 (20.4)
	ST	37 (19.9)
	OBC	111 (59.7)
Residence	<10 years	86 (46.2)
	≥10 years	100 (53.8)
Educational status	Illiterate	29 (15.6)
	Primary	28 (15.0)
	Middle school	92 (49.5)
	High school and above	37 (19.9)
Occupation	Unemployed	111 (59.7)
	Unskilled worker	20 (10.8)
	Semiskilled worker	35 (18.8)
	Skilled worker	11 (5.9)
	Clerk/shop owner/farm owner	6 (3.2)
	Semiprofessional	3 (1.6)
	Professional	0 (0.0)
Socioeconomic status	Class I	35 (18.8)
	Class II	40 (21.5)
	Class III	65 (34.9)
	Class IV	33 (17.8)
	Class V	13 (7.0)
Type of family	Nuclear	108 (58.1)
	Joint	33 (17.7)
	Three-generation	45 (24.2)
Trimester of pregnancy	First	56 (30.1)
	Second	112 (60.2)
	Third	18 (9.7)
Gravida	Primigravida	114 (61.3)
	Gravida 2	44 (23.7)
	Gravida ≥3	28 (15.1)

Palpebral conjunctival and nail bed pallor were each observed in 140 (75.2%) participants, followed by palmar pallor in 110 (59.1%) participants and palmar crease pallor in 74 (39.8%) participants (Table 2).

**Table 2:** Distribution of Clinical Pallor at Different Anatomical Sites Among the Study Participants (N = 186)

Site	Clinical Pallor	n (%)
Palpebral conjunctiva	Present	140 (75.2)
	Absent	46 (24.8)
Nail bed	Present	140 (75.2)
	Absent	46 (24.8)
Palm	Present	110 (59.1)
	Absent	76 (40.9)
Palmar crease	Present	74 (39.8)
	Absent	112 (60.2)

The overall prevalence of anemia among pregnant women in this study was 139 (74.7%), as determined by HemoCue® hemoglobin estimation. Among the 186 participants, 47 (25.3%) had normal hemoglobin levels, 61 (32.8%) had mild anemia, 60 (32.2%) had moderate anemia, and 18 (9.7%) had severe anemia. The distribution of anemia severity based on palpebral conjunctival pallor closely resembled that obtained by HemoCue® hemoglobin estimation, with most participants classified as having mild or moderate anemia. Nail bed pallor showed a similar distribution. In contrast, palm pallor classified a higher proportion of participants as having normal hemoglobin levels or severe anemia and a lower proportion as having mild anemia than the HemoCue® reference standard. Palmar crease pallor showed the greatest discrepancy, classifying the highest proportion of participants as having normal hemoglobin levels and the lowest proportion as having mild anemia compared with HemoCue® hemoglobin estimation (Table 3).

**Table 3:** Comparison of Anemia Severity Based on Clinical Pallor at Different Anatomical Sites and HemoCue® Hemoglobin Estimation (Reference Standard) (N = 186)

Grades of Anemia	Palpebral Conjunctiva n (%)	Nail Bed n (%)	Palm n(%)	Palmar Crease n (%)	HemoCue® (Reference Standard) n(%)
Normal	46 (24.7)	46 (24.7)	76 (40.8)	112 (60.2)	47 (25.3)
Mild	71 (38.2)	70 (37.6)	38 (20.4)	15 (8.1)	61 (32.8)
Moderate	54 (29.0)	49 (26.3)	42 (22.6)	34 (18.3)	60 (32.2)
Severe	15 (8.1)	21 (11.3)	30 (16.1)	25 (13.4)	18 (9.7)
Total	186 (100)	186 (100)	186 (100)	186 (100)	186 (100)

The highest percentage agreement was observed for palpebral conjunctival pallor (93.3%; Cohen's κ = 0.86), followed by the nail bed (80.0%; κ = 0.62), palm (76.7%; κ = 0.58), and palmar crease (66.7%; κ = 0.40). These findings indicate that palpebral conjunctival pallor demonstrated the highest inter-observer reliability among the anatomical sites evaluated (Table 4).

**Table 4:** Inter-observer Agreement in Detection of Pallor at Different Anatomical Sites (N = 30) N = 30 denotes the first 30 participants included in the study for the initial inter-observer reliability assessment. These participants were also included in the final analysis.

Anatomical Site	Observer 1 Positive n (%)	Observer 2 Positive n (%)	Percentage Agreement (%)	Kappa Value
Palpebral conjunctiva	23 (76.7%)	24 (80.0%)	93.30%	0.86
Nail bed	22 (73.3%)	24 (80.0%)	80.00%	0.62
Palm	18 (60.0%)	20 (66.7%)	76.70%	0.58
Palmar crease	12 (40.0%)	14 (46.7%)	66.70%	0.4

The 2 × 2 contingency tables comparing clinical pallor at different anatomical sites with the HemoCue® reference standard are presented in Table 5. Palpebral conjunctival pallor showed the highest number of true positives and the fewest false negatives, whereas palmar crease pallor showed the greatest number of false negatives.

**Table 5:** 2 × 2 Contingency Tables for Clinical Pallor at Different Anatomical Sites Compared With HemoCue® Hemoglobin Estimation (Reference Standard) (N = 186) TP: true positive (clinical pallor present and anemia confirmed by HemoCue); FP: false positive (clinical pallor present but no anemia by HemoCue®); FN: false negative (clinical pallor absent despite anemia by HemoCue®); TN: true negative (clinical pallor absent and no anemia by HemoCue). HemoCue® hemoglobin estimation was used as the reference standard for diagnosing anemia.

Anatomical Site	TP	FP	FN	TN
Palpebral conjunctiva	137	3	2	44
Nail bed	113	27	26	20
Palm	100	10	39	37
Palmar crease	64	10	75	37

Palpebral conjunctival pallor demonstrated the highest diagnostic accuracy, with a sensitivity of 98.6%, specificity of 93.6%, overall agreement of 97.3%, and a kappa value of 0.92. Nail bed pallor showed a sensitivity of 81.3% and a specificity of 42.6%, with an overall agreement of 71.5% (κ = 0.63). Palmar pallor had a sensitivity of 71.9%, specificity of 78.7%, and an agreement of 73.6% (κ = 0.62). Palmar crease pallor demonstrated the lowest sensitivity (46.0%) but a specificity of 78.7%, with an overall agreement of 54.3% and a kappa value of 0.30.

Palpebral conjunctival pallor demonstrated the highest LR+ (15.45) and the lowest LR- (0.02). Palm, palmar crease, and nail bed pallor showed lower LR+ (3.38, 2.16, and 1.41, respectively) and higher LR- (0.36, 0.69, and 0.44, respectively) (Table 6).

**Table 6:** Diagnostic Accuracy and Agreement of Pallor at Different Anatomical Sites Compared With HemoCue® Hemoglobin Estimation for the Detection of Anemia (N = 186) PPV: positive predictive value; NPV: negative predictive value; CI: confidence interval; LR+: positive likelihood ratio; LR−: negative likelihood ratio; κ: Cohen's kappa coefficient.

Anatomical Site	Sensitivity % (95% CI)	Specificity % (95% CI)	PPV % (95% CI)	NPV % (95% CI)	LR+	LR−	Agreement (%)	Cohen's k
Palpebral conjunctiva	98.56 (94.9–99.8)	93.62 (82.5–98.7)	97.86 (93.9–99.5)	95.65 (84.9–99.5)	15.45	0.02	97.3	0.92
Nail bed	81.29 (73.8–87.4)	42.55 (28.3–57.8)	80.71 (73.2–86.8)	43.48 (29.8–57.8)	3.38	0.36	71.5	0.63
Palm	71.94 (63.8–79.1)	78.72 (64.3–89.3)	90.91 (84.0–95.5)	48.68 (37.0–60.5)	2.16	0.69	73.6	0.62
Palmar crease	46.04 (37.6–54.7)	78.72 (64.3–89.3)	86.49 (76.5–93.3)	33.04 (24.4–42.6)	1.41	0.44	54.3	0.3

## Discussion

The present study demonstrated a high prevalence of anemia (74.7%) among pregnant women attending a rural primary healthcare center. This prevalence is higher than that reported by the NFHS-5, which documented anemia among 52.2% of pregnant women in India and 42.2% in Delhi. Similar high prevalence rates have been reported in other Indian studies, including that by Suryanarayana R et al., who highlighted anemia during pregnancy as a persistent public health challenge requiring effective and affordable screening strategies at the primary care level [16].

Among the four anatomical sites evaluated, palpebral conjunctival pallor demonstrated the highest diagnostic accuracy, with a sensitivity of 98.56%, a specificity of 93.62%, excellent agreement with HemoCue® (κ = 0.92), the highest LR+ (15.45), and the lowest LR- (0.02). These findings indicate that conjunctival pallor is highly effective both in identifying women with anemia and in excluding those without anemia. Similar observations have been reported by Chalco JP et al. [17] and Sheth TN et al. [11], who identified conjunctival pallor as the most reliable clinical sign for detecting anemia. The superior performance of conjunctival pallor may be explained by the rich vascularity and thin mucosal surface of the conjunctiva, where reductions in hemoglobin concentration become readily apparent and are less influenced by skin pigmentation than pallor at other anatomical sites. These findings also support the WHO recommendation that pallor assessment should remain an integral component of routine antenatal examination, particularly in resource-limited settings where hemoglobin estimation may not be immediately available.

Nail bed pallor demonstrated good sensitivity (81.29%) but poor specificity (42.55%), indicating that although most anemic women were correctly identified, a considerable proportion of non-anemic women were also classified as having pallor. Similar findings were reported by Nardone DA et al. [18], who suggested that variations in peripheral perfusion, capillary refill, and observer interpretation may reduce the reliability of nail bed examination. Therefore, nail bed pallor may be useful as an adjunctive clinical sign but is less suitable as a standalone screening method.

Palmar pallor showed moderate sensitivity (71.94%) and specificity (78.72%), indicating balanced but lower diagnostic performance than conjunctival pallor. These findings are consistent with those of Kalantri A et al. [13], who also reported moderate diagnostic accuracy for palmar pallor. Although examination of the palm is simple and practical during routine antenatal care, its lower sensitivity suggests that a proportion of anemic women may remain undetected if it is used as the sole screening method.

Among all the anatomical sites examined, palmar crease pallor demonstrated the lowest sensitivity (46.04%) despite moderate specificity (78.72%), indicating limited usefulness as a screening test for anemia. The lower sensitivity suggests that reliance on palmar crease pallor alone would result in a substantial number of missed cases. Similar observations have been reported in previous studies, where palmar crease pallor was found to be less reliable than conjunctival examination for identifying anemia [11, 12].

Overall, the findings of the present study are consistent with previous investigations evaluating the diagnostic utility of clinical pallor. Bergsjø P et al. [12] demonstrated that although the diagnostic accuracy of clinical pallor varies across anatomical sites, it remains a practical and valuable screening method during pregnancy, particularly in low-resource settings. Likewise, Butt Z et al. [19] reported that conjunctival pallor has superior diagnostic performance compared with other clinical signs. The present study extends these observations by directly comparing four commonly examined anatomical sites within the same population using HemoCue® hemoglobin estimation as the reference standard.

Inter-observer agreement further strengthened the reliability of the findings. Palpebral conjunctival pallor demonstrated the highest reproducibility (κ = 0.86), whereas palmar crease pallor showed the lowest agreement (κ = 0.40). These findings indicate that conjunctival pallor is not only the most accurate but also the most reproducible clinical sign, suggesting that it can be consistently assessed by trained healthcare workers in routine antenatal practice.

From a clinical perspective, physical examination should be regarded as a screening rather than a definitive diagnostic method. Guyatt GH et al. [20] emphasized that laboratory hemoglobin estimation remains the reference standard for diagnosing anemia, while clinical assessment provides valuable preliminary information, particularly in settings where laboratory facilities are unavailable. The present findings reinforce this concept by demonstrating that palpebral conjunctival pallor has excellent diagnostic accuracy and can effectively identify women requiring confirmatory hemoglobin estimation or early treatment.

The findings of this study have important implications for primary healthcare practice. Routine assessment of palpebral conjunctival pallor should be incorporated into standard antenatal screening protocols because of its high sensitivity, specificity, reproducibility, and overall diagnostic accuracy. Examination of the nail bed, palm, and palmar crease may provide complementary clinical information but should not replace conjunctival assessment owing to their comparatively lower diagnostic performance. Incorporating standardized conjunctival examination into routine antenatal care could facilitate early detection and timely management of anemia while reducing dependence on laboratory infrastructure in resource-constrained settings.

Study strengths

This study provides a comprehensive comparison of the diagnostic accuracy of clinical pallor at four anatomical sites using HemoCue® hemoglobin estimation as the reference standard among pregnant women. Standardized operational definitions and a uniform examination protocol under natural light improved the consistency of clinical assessment. Inter-observer agreement was also evaluated, demonstrating good reproducibility, particularly for conjunctival pallor. In addition, multiple diagnostic accuracy measures, including sensitivity, specificity, predictive values, LRs, and Cohen's κ, were reported, providing a robust evaluation of the clinical utility of each anatomical site. The findings are directly applicable to primary healthcare settings where access to laboratory facilities may be limited.

Study limitations

The study was conducted at a single rural primary health center with a relatively small sample size, which may limit the generalizability of the findings to other populations and healthcare settings. Consecutive sampling may have introduced selection bias. Although standardized assessment and observer training were undertaken, clinical evaluation of pallor remains subjective and may be influenced by factors such as skin pigmentation, ambient lighting, and examiner experience. Furthermore, HemoCue®, although a validated point-of-care device, was used as the reference standard instead of an automated laboratory hematology analyzer. Future multicentric studies with larger sample sizes are warranted to validate these findings across diverse populations.

## Conclusions

Anemia was common among pregnant women in this study. Among the clinical signs evaluated, palpebral conjunctival pallor demonstrated the highest diagnostic accuracy and showed excellent agreement with HemoCue® hemoglobin estimation, making it the most reliable clinical indicator for screening anemia in primary care settings. Nail bed pallor demonstrated good sensitivity but limited specificity, whereas palm pallor demonstrated moderate diagnostic performance. Palmar crease pallor was the least sensitive clinical sign. Overall, assessment of palpebral conjunctival pallor is a simple, feasible, and effective method for screening anemia in resource-limited primary care settings where hemoglobin estimation is not readily available.

## Appendices

Appendix A

Study Proforma

**Table 7:** Study Proforma: Participant Information, Obstetric Details, and Eligibility Screening

Variable	Response
Participant Information	
Participant ID	
Date of Interview	____ / ____ / ______
Time	____ : ____ AM/PM
Age (completed years)	
Residence	□ Barwala □ Adjacent village
Duration of residence	□ <10 years □ ≥10 years
Religion	□ Hindu □ Muslim □ Christian □ Other ___________
Caste	□ SC □ ST □ OBC □ Other _______________
Educational status	□ Illiterate □ Primary □ Middle School □ High School & Above
Occupation	□ Unemployed □ Unskilled worker □ Semiskilled worker □ Skilled worker □ Clerk/Shop Owner/Farm Owner □ Semi-professional □ Professional
Per capita monthly family income (₹)	
Socioeconomic status (Modified B.G. Prasad Scale 2024)	□ Class I □ Class II □ Class III □ Class IV □ Class V
Type of family	□ Nuclear □ Joint □ Three-generation
Obstetric Details	
Gravidity	□ Primigravida □ Gravida 2 □ Gravida ≥3
Gestational age	______ weeks
Trimester	□ First □ Second □ Third
Eligibility Screening	
Mehendi (henna) on palms or fingers	□ Yes □ No
Nail polish/artificial nails	□ Yes □ No
Active skin disease/conjunctivitis	□ Yes □ No
Acute illness affecting peripheral perfusion	□ Yes □ No

**Table 8:** Clinical Pallor Assessment (Index Test)

Anatomical Site	Operational Definition	Present	Absent
Palpebral conjunctiva	Pallor present when the anterior rim of the lower palpebral conjunctiva appears as pale as the deeper posterior conjunctiva.	□	□
Nail bed	Pallor present when persistent pallor is observed after gentle pressure release.	□	□
Palm	Pallor present when generalized paleness of the palm is observed under adequate natural light.	□	□
Palmar crease	Pallor present when the palmar creases appear as pale as or paler than the surrounding skin.	□	□

**Table 9:** Reference Standard (HemoCue® Hb 201)

Variable	Response
Date of Hb Estimation	________ / _______ / __________
Time	____ : ____ AM/PM
Blood Sample	Capillary blood
Finger Used	□ Right □ Left
HemoCue® Hb (g/dL)	
Anemia (Hb <11.0 g/dL)	□ Yes □ No
WHO Anemia Classification	□ Normal (Hb ≥11.0 g/dL) □ Mild Anemia (Hb 10.0–10.9 g/dL) □ Moderate Anemia (Hb 7.0–9.9 g/dL) □ Severe Anemia (Hb <7.0 g/dL)

**Table 10:** Inter-Observer Assessment of Pallor at Different Anatomical Sites Among a Subset of Participants (N = 30)

Anatomical Site	Observer 1	Observer 2	Agreement
Palpebral conjunctiva	□ Present □ Absent	□ Present □ Absent	□ Yes □ No
Nail bed	□ Present □ Absent	□ Present □ Absent	□ Yes □ No
Palm	□ Present □ Absent	□ Present □ Absent	□ Yes □ No
Palmar crease	□ Present □ Absent	□ Present □ Absent	□ Yes □ No

**Table 11:** Observer Assessment Details

Variable	Response
Observer 1 Initials	
Observer 2 Initials	
Observers blinded to each other's findings	□ Yes □ No
Observers blinded to HemoCue® result	□ Yes □ No
Time interval between examinations (minutes)	

## References

[1] (2026). Hemoglobin concentrations for the diagnosis of anemia and assessment of severity. https://www.who.int/publications/i/item/WHO-NMH-NHD-MNM-11.1.

[2] (2026). Anaemia in women and children. https://www.who.int/data/gho/data/themes/topics/anaemia_in_women_and_children.

[3] (2026). The global prevalence of anaemia in 2011. https://www.who.int/publications/i/item/9789241564960.

[4] (2026). India: National Family Health Survey (NFHS-4), 2015-16. https://dhsprogram.com/pubs/pdf/FR339/FR339.pdf.

[5] (2026). India: National Family Health Survey (NFHS-5), 2019-21. https://dhsprogram.com/pubs/pdf/FR375/FR375.pdf.

[6] (2026). Anaemia Mukt Bharat, Poshan Abhiyan. https://anemiamuktbharat.info/.

[7] Gadapani Pathak B, Vats P, Jadaun A, Garg N, Mazumder S (2026). Implementation challenges of India's national anaemia reduction program among pregnant women: insights from mixed-methods implementation research. Front Glob Womens Health.

[8] (2026). Pregnancy, childbirth, postpartum and newborn care: a guide for essential practice (3rd edition). https://www.who.int/publications/i/item/9789241549356.

[9] Horibata K, Kondo S, Hashimoto S, Takemura Y (2025). An observational study to determine the optimal physical evaluation site for detecting anemia. J Gen Fam Med.

[10] Powers JM, Brandow AM (2023). Pallor and anemia. Nelson Pediatric Symptom-Based Diagnosis: Common Diseases and their Mimics (Second Edition).

[11] Sheth TN, Choudhry NK, Bowes M, Detsky AS (1997). The relation of conjunctival pallor to the presence of anemia. J Gen Intern Med.

[12] Bergsjø P, Evjen-Olsen B, Hinderaker SG, Oleking'ori N, Klepp KI (2008). Validity of non-invasive assessment of anaemia in pregnancy. Trop Med Int Health.

[13] Kalantri A, Karambelkar M, Joshi R, Kalantri S, Jajoo U (2010). Accuracy and reliability of pallor for detecting anaemia: a hospital-based diagnostic accuracy study. PLoS One.

[14] Bala DV, Vyas S, Shukla A, Tiwari H, Bhatt G, Gupta K (2012). Validity and reliability of haemoglobin colour scale and its comparison with clinical signs in diagnosing anaemia in pregnancy in Ahmedabad, India. East Mediterr Health J.

[15] K J A, Mishra A, Borle AL (2024). Updated B. G. Prasad scale for socioeconomic status classification for the year 2024. Indian J Pediatr.

[16] Suryanarayana R, Santhuram AN, Chandrappa M, Shivajirao P, Rangappa SS (2016). Prevalence of anaemia among pregnant women in rural population of Kolar district. Int J Med Sci Public Health.

[17] Chalco JP, Huicho L, Alamo C, Carreazo NY, Bada CA (2005). Accuracy of clinical pallor in the diagnosis of anaemia in children: a meta-analysis. BMC Pediatr.

[18] Nardone DA, Roth KM, Mazur DJ, McAfee JH (1990). Usefulness of physical examination in detecting the presence or absence of anemia. Arch Intern Med.

[19] Butt Z, Ashfaq U, Sherazi SF, Jan NU, Shahbaz U (2010). Diagnostic accuracy of "pallor" for detecting mild and severe anaemia in hospitalized patients. J Pak Med Assoc.

[20] Guyatt GH, Oxman AD, Ali M, Willan A, McIlroy W, Patterson C (1992). Laboratory diagnosis of iron-deficiency anemia: an overview. J Gen Intern Med.

